# Genetic variability of histamine receptors in patients with Parkinson's disease

**DOI:** 10.1186/1471-2350-9-15

**Published:** 2008-03-17

**Authors:** Elena García-Martín, P Ayuso, Antonio Luengo, Carmen Martínez, José AG Agúndez

**Affiliations:** 1 Department of Biochemistry and Molecular Biology, School of Biological Sciences, University of Extremadura, Badajoz, Spain; 2 Service of Neurology, La Princesa University Hospital, Madrid, Spain; 3 Department of Pharmacology & Psychiatry, Medical School, University of Extremadura, Badajoz, Spain

## Abstract

**Background:**

Changes in the density and expression of histamine receptors (HRH) have been detected in Parkinson's disease (PD) patients, and HRH antagonists bring about improvements in motor and other symptoms, thus suggesting that HRH play a role in the clinical response of PD patients. This study is aimed to analyse polymorphic variations of HRH in patients with PD.

**Methods:**

Leukocytary DNA from 195 PD patients and a control group of 231 unrelated healthy individuals was studied for the nonsynonymous HRH1Leu449Ser and the promoter HRH2G-1018A polymorphisms by using amplification-restriction analyses.

**Results:**

The HRH1Leu449Ser amino acid substitution was identified in two women with late-onset PD whereas it was not observed among healthy subjects. The HRH2G-1018A polymorphism was observed with allele frequencies = 3.59 (95% CI = 1.74–5.44) and 5.0 (95% CI = 3.00–6.96) for patients with PD and healthy controls, respectively. These frequencies were independent of gender and age of onset of the disease. Multiple comparison analyses revealed that differences were not statistically significant.

**Conclusion:**

These results indicate that the polymorphisms analyzed are not a major risk factor for PD, although the HRH1Leu449Ser amino acid substitution might be related to PD.

## Background

Histamine is involved in neuronal degeneration [1] and neurotoxicity [2]. Changes in the morphology and increase in density of histaminergic fibers in the substantia nigra have been described in the brain of PD patients [3]. It has been shown that histamine causes selective damage in the dopaminergic neurons of the substantia nigra with induction of inflammatory signal processes [4]. Among patients with PD, blood histamine levels [5] and the concentration of the histamine metabolite pros-methylimidazoleatic acid in the cerebrospinal fluid are increased [6], and recently we described the association of genotypes leading to high histamine metabolism with increased risk to develop PD [7]. Taken together, these findings suggest that modulation of brain histamine may be related to PD, but the mechanisms underlying such modulation remains unknown.

Changes in the density and expression of histamine receptors (HRH) have been detected in PD patients [8]. HRH antagonists bring about improvements in motor and other symptoms [9,10], thus suggesting that HRH play a role in the clinical response of PD patients. Since genes coding for HRH are polymorphic it may be speculated that variations in genes coding for HRH could be related to PD.

Three receptors, HRH1 through HRH3 are present in the human brain. HRH1 and HRH2 have been localized to caudate and putamen, and HRH3 is most abundant in the basal ganglia, the highest density being observed in the globus pallidus [11]. The predominant location of HRH2 and HRH3 in the basal ganglia suggests that these receptors could play a role in motor functions [11]. In addition, it has recently been shown that a protective role against toxic-induced neurodegeneration is linked to expression of histamine receptors [12]. Taking together the alteration in histaminergic transmission observed in patients with PD, the fact that brain histamine receptors are believed to regulate the release of brain histamine and other neurotransmitters [13-16] and the putative role of histamine receptors in motor functions, it can be speculated that individuals carrying mutated histamine receptors may show altered susceptibility to develop PD.

Studies involving single nucleotide polymorphisms (SNP) in HRH in PD patients are scarce and controversial. HRH1 and HRH2 are polymorphic due to genetic alterations, whereas no variant HRH3 alleles have been described so far, with the exception of a rare Ala280Val described in a patient suffering from Shy-Drager syndrome which was not identified in other subjects [17]. The *HRH1 *gene, located in chromosome 3p25, shows diverse SNPs, and one of these, designated as rs2067470, located in exon 3 T1522C, causes the amino acid substitution Leu449Ser [18]. Other *HRH1 *variant alleles causing amino acid changes at positions 19, 270, 308 and 349 have been described [19], but none of these have been identified in Caucasian individuals [20]. Among the SNPs mapped to the *HRH2 *gene, located in chromosome 5q35.2, in addition to the variant *HRH2 *allele causing the Asn217Asp amino acid change, initially described by Orange et al [21,22] but not confirmed by independent studies [19,23], other variant *HRH2 *alleles have been described, although none of these cause amino acid changes [19,24]. Only one SNP, designated as rs2067474, a G-1018A transition located in an enhancer element of the gene promoter, occurs with a relevant allele frequency [18].

It can be hypothesized that genetic variations in histamine receptors may modulate the risk to develop PD because polymorphisms at histamine receptors are likely to have relevant functional effects, although the functional characterization for histamine receptor polymorphisms has not been completed so far. For instance, it has been shown that the HRH1 Leu449Ser is related to the risk to develop schizophrenia. Although the mechanism involved in such association remains to be elucidated [18], the presence of serine, which is more hydrophillic and reactive than leucine, is likely to induce functional changes in the receptor. The other SNPs tested at the HRH1 gene, namely Gly270Glu, induces a change from a small to a medium-size and acidic amino acid. The HRH2 SNP occurs in an enhancer element of the gene promoter [23]. It is likely that the HRH2 variant located in the promoter may induce changes in the expression of receptors. This study is aimed to test the hypothesis that genetic alterations in histamine receptors, either causing changes in the expression or functional alterations related to amino acid substitutions, may cause an increased risk to develop PD. The HRH gene products may have functional relevance to PD as they encode histamine receptors, and hence may influence histamine effects on CNS. In an attempt to identify factors related to PD risk, we mapped common SNPs in the *HRH1 *and *HRH2 *genes in patients with PD and healthy subjects.

## Methods

All the participants were Caucasian Spanish individuals and were included in the study after giving informed consent. We studied a cohort of 195 patients suffering from sporadic PD who presented to the neurological service in la Princesa Hospital, Madrid, Spain. All consecutive patients diagnosed by consultant neurologists according to the criteria recommended by Hughes et al. [25] were requested to participate, and all of them agreed. Healthy subjects (n = 231) were randomly selected among medical students, and university and hospital staff. A medical examination was performed to identify subjects in good health. Over 95% of the healthy subjects propositioned agreed to participate. All lived in the same areas as the patients (Madrid and surrounding areas). The protocol of the study was approved by the Ethics Committees of the hospitals involved in the study. Table 1 summarizes the demographic data of the subgroups analyzed in the study. To test for a putative age-related difference in the frequency for the SNPs analyzed, we examined the frequencies of HRH variant alleles in a group of 41 healthy Spanish nonagenarians free of neurodegenerative diseases who had participated in previous genetics studies [26].

**Table 1:** Characteristics of the individuals included in the study.

	PATIENTS WITH PD (%)	HEALTHY SUBJECTS (%)
Men	93 (47.7)	110 (47.6)
Women	102 (52.3)	121 (52.3)
Age ± sd (range)	69.3 ± 22.0 (28–90)	28.9 ± 12.5 (19–84)

Genomic DNA was obtained from peripheral leukocytes and purified according to standard procedures. The presence of the SPNs was investigated by amplification-restriction and electrophoresis in agarose gels. In order to prevent false-positive or negative findings due to lack of endonuclease digestions, the detection methods were designed including both constitutive and polymorphic restriction sites for the two SNPs analyzed. The analysis of the *HRH1 *Gly270Glu rs7651620 polymorphism was carried out after the amplification of a gene fragment of exon 3 by using the following primers (based on the published human HRH1 sequence Gene Bank Accession No. NM_000861): TTC ATG CAG CAG ACC TCG GTG and AGG CCC TGC TCA TCT GTC TTG. After an initial step of 3 minutes at 94°C, PCR amplification was carried out for 40 cycles of 25 sec at 94°C, 1 minute at 56°C, and 1 minute at 72°C, and a final extension period of 5 minutes at 72°C. The 512 bp PCR product contains a constitutive restriction site for the endonuclease *Hin*fI, and a variant-allele specific restriction site. After endonuclease digestion the wild-type gene yielded fragments of 259 and 253 bp, whereas the mutated gene was digested to fragments of 259, 205 and 48 bp. No variant alleles for the rs7651620 polymorphism were detected among PD patients or control subjects.

The analysis of the *HRH1 *Leu449Ser rs2067470 polymorphism was carried out after the amplification of a gene fragment by using the primers (based on the published human HRH1 sequence Gene Bank Accession No. NM_000861): CGAACGGACTCAGATACCACC and CTGGCAACACACAGGCCTTC. After an initial step of 3 minutes at 94°C, PCR amplification was carried out for 40 cycles of 25 sec at 94°C, 1 minute at 55°C, and 1 minute at 72°C, and a final extension period of 5 minutes at 72°C. The 470 bp PCR product was sequenced as follows: The sequencing mixture contained 2 μl of the purified PCR products and 60 nM of the corresponding primer, and was assembled according the instructions of the manufacturer (dRhodamine terminator cycle sequencing kit, Applied Biosystems). Automated sequencing was carried out in an Abi Prism 310 genetic analyzer (Applied Biosystems). The sequencing conditions were as described elsewhere [27].

Regarding the G-1018A transition in the *HRH2 *gene, the following primers (based on the HRH2 gene sequence Gene Bank Accession No. AB023486) were used: ACA GCC CGT GGC TAA GAA TGG and AGA AGG GAG GCA GGA TGC AAG. After an initial step of 3 minutes at 94°C, PCR amplification was carried out for 40 cycles of 25 sec at 94°C, 1 minute at 62°C, and 1 minute at 72°C, and a final extension period of 5 minutes at 72°C. The amplified 574 bp contained three constitutive restriction sites for the endonuclease *BsuR*I, yielding fragments of 262, 148, 125 and 39 bp. One of the restriction sites was lost in the variant allele which after digestion yielded fragments of 410, 125 and 39 bp. Control DNA samples with known sequences that were wild-type, heterozygous and homozygous for every SPN analyzed were ran in parallel to ensure accurate genotyping.

The statistical power with the sample size finally involved in the study (n = 195) was 98.5% for the *HRH1 *variant allele and 98.1% for the for the *HRH2 *variant allele (unilateral association). The frequencies for the *HRH *were estimated by counting genes and calculating sample proportions. Case-control analyses were performed with the χ 2 statistics or Fisher exact test, each when appropriate. The association between *HRH *polymorphisms and gender or age of onset of PD was estimated by odds ratio (OR) with 95% confidence interval (CI). To assess whether allelic variants influence synergically upon the age of onset of PD, subjects were classified as carriers and noncarriers of nonsynonymous SNPs. The above-cited test was used for the comparison of carriers and noncarriers. Logistic regression analysis was performed to assess whether *HRH *gene polymorphisms were correlated with gender-related risk or age of onset. Association was expressed as OR with 95% CI. Statistical analysis was performed using the Statistical Package for the Social Sciences (SPSS) version 10.07 for Windows (SPSS Inc., Chicago, Ill. USA). Statistical analyses were adjusted for multiple comparisons by the use of Bonferroni's test. A two-tailed p value equal to or less than 0.01 was considered significant.

## Results

The fragments of both histamine receptor genes containing the polymorphisms to be analyzed were amplified in all participants, and in all cases the products of gene amplifications had identical sizes. Table 2 shows the genotypes of PD patients and controls. Individuals carrying the *HRH1 *SNP were identified among PD patients, but not in the control group. Regarding *HRH2 *genotypes, a lower frequency for variant alleles was observed among PD patients, although the differences were not statistically significant. Both patients and controls were in Hardy-Weinberg equilibrium for the *HRH1 *and *HRH2 *genotypes, with expected frequencies for subjects with the wild-type, heterozygous and homozygous genotype as follows: *HRH1 *genotype = 99.0%, 1.0% and 0% for patients and 100.0%, 0% and 0% for healthy subjects; *HRH2 *genotype = 92.9%, 6.9% and 0% for patients and 90.3%, 9.5% and 0.2% for control subjects. These expected frequencies are close to the actual frequencies shown in Table 2. To test for putative age-related differences for allele frequencies in the control population, we analyzed both polymorphisms in nonagenarians. The allele frequencies among nonagenarian subjects were identical to those present in the group of 231 healthy individuals: 0% and 4.9% of variant alleles for the *HRH1 *and *HRH2 *polymorphisms, respectively. No gender or age-related differences in genotypes or allele frequencies were observed in the control group.

**Table 2:** Histamine receptor polymorphisms in patients with PD and healthy controls.

GENOTYPE	PATIENTS WITH PD NO (%)	HEALTHY SUBJECTS NO (%)	INTERGROUP COMPARISON VALUES ODDS RATIO (95% C.I) [CHI-SQUARE; P VALUE]
H1 Leu/Leu	193 (99.0)	231 (100)	0.46^a ^(0.46–1.34) [2.38; 0.123]
H1 Leu/Ser	2 (1.0)	0 ---	2.20 ^a ^(0.75–2.20) [2.38; 0.123]
H1 Ser/Ser	0 ---	0 ---	---
H2 -1018 G/G	183 (93.9)	208 (90.0)	1.69 (0.83–3.44) [2.03; 0.154]
H2 -1018 G/A	10 (5.1)	23 (10.0)	0.49 (0.23–1.04) [3.44; 0.063]
H2 -1018 A/A	2 (1.0)	0 ---	2.20 ^a ^(0.75–2.20) [2.38; 0.123]
**Total**	**195**	**231**	
*HRH1 *Allele frequency			
H1 Leu	388 (99.5)	462 (100)	0.46 ^a ^(0.46–1.34) [2.37; 0.124]
H1 Ser	2 (0.5)	0 (0)	2.19 ^a ^(0.74–2.19) [2.37; 0.124]
**Total**	**390**	**462**	
*HRH2 *Allele frequency			
H2 -1018 G	376 (96.4)	439 (95.0)	1.41 (0.72–2.74) [0.98; 0.322]
H2 -1018 A	14 (0.6)	23 (5.0)	0.71 (0.37–1.39) [0.98; 0.322]
**Total**	**390**	**462**	

Crude Odds ratios are shown. (^a^) In cases where comparison values included zero the relative risk ratio is shown instead of the Odds Ratio.

Patients were subdivided into groups according to gender and age of onset of symptoms. Table 3 shows that variant *HRH1 *alleles are observed among women with late-onset PD. However, because of the low frequency for these SNPs, the results were not statistically significant for multiple comparison values. No major differences regarding gender or age of onset were observed among PD patients with regard to the *HRH2 *polymorphism.

**Table 3:** Histamine receptor polymorphisms according to gender and age of onset of PD. PD patients were subdivided in groups according to age of onset (under or over the median age) and according to gender.

	PD PATIENTS *H1 *SER N (OR; 95 C.I.) [Χ^2^; P VALUE]	PD PATIENTS *H2 *– 1018 A N (OR; 95 C.I.) [Χ^2^; P VALUE]	CONTROL SUBJECTS *H2 *– 1018 A N
Overall patients	2 (2.19 ^a^; 0.74–2.19) [2.37,0.123]	14 (0.71; 0.37–1.39) [0.98, 0.322]	23
Men	0, (--)	3 (0.28; 0.08–0.95) [4.17,0.041]	12
Women	2 (2.19 ^a^; 0.74–2.20) [2.37,0.123]	11 (1.20; 0.52–2.76) [0.16, 0.681]	11
Onset under 68 yr	0, (--)	6 (0.58; 0.24–1.42) [1.36, 0.244]	N.A.
Onset over 68 yr	2 (3.45 ^a^; 1.19–3.48) (4.92;0.027)	8 (0.85; 0.38–1.90) [0.154, 0.695]	N.A.

(^a^) In cases where comparison values included zero the relative risk ratio is shown instead of the Odds Ratio.

N.A. Not applicable.

Chi-square and *p *values correspond to a Mantel-Haenszel comparison with healthy individuals.

## Discussion

The etiology of idiopathic PD is complex and poorly understood, although epidemiological data and animal models involve both genetic and environmental factors in the etiology of the disease [28-31]. Initially promising findings of genomewide association analyses [32] were not confirmed by further studies [33-37]. Nevertheless, evidence of an inheritance role is still supported by the fact that 6% to 33% of PD patients have relatives with the disease [38-41]. In the present study we investigated the role of polymorphisms of histamine-receptors in PD. The rationale for the study is that histamine seems to play a role in PD and that among the three histamine receptors present in the brain, two of them, namely HRH1 and HRH2 are polymorphic.

It should be emphasized that in the present study patients and controls were not age-matched. Nevertheless, we observed that age-related differences in allele frequencies do not occur in healthy subjects. It cannot be ruled out that some control individuals would eventually develop PD. Nevertheless, the possibility that some healthy subject would eventually develop PD in the lapse between the mean age of controls and the mean age of cases is negligible given the prevalence of PD (<1%) at ages less than 68 years in the studied population [42], and therefore the differences in the mean age of patients and controls should not influence the findings obtained in the present study. The *HRH1 *variant allele frequency in our study population is extremely low. We have demonstrated the occurrence of the Leu449Ser HRH1 amino acid polymorphism in two Caucasian women with PD, but this variant allele was not detected in 231 healthy subjects. Such low frequency is in contrast with the allele frequency initially reported for the variant allele [43], but it agrees with the absence of such variant allele in 58 European individuals that participated in the Hap-Map project [44]. The *HRH2 *variant allele frequency in our population study, as calculated from Table 2, is 3.5% in PD patients and 4.9% in healthy subjects. Such frequency is lower as that initially reported [23]. Although the occurrence of ethnic differences between different Caucasian subjects such as Swedish, French or Spanish individuals regarding HRH variant alleles has not been investigated in detail such differences may occur, as it has been demonstrated for common SNPs on genes coding for drug-metabolizing enzymes [45]. Another possible explanation for differences in *HRH2 *allele frequencies between the present study and that by Ito et al. [23] is related to sampling. In our study we analyzed 231 unrelated healthy subjects, whereas in the study by Ito et al. [23] authors analyze only 53 Swedish subjects and 52 French subjects. In addition, the Swedish subjects such study belonged to 15 families and therefore these were not unrelated subjects. The combination of the low sample size and the fact that some participants are relatives may cause a bias in allele frequencies. In addition, it should be mentioned that our findings agree with the presence of such variant allele with a frequency of 4.2% in 60 European individuals that participated in the Hap-Map project [46], which is very close to the frequency of 4.9% reported here (Table 3). In summary, the findings obtained in the present study do not support the high allele frequencies initially reported for the variant alleles analyzed [23,43]. The design of the amplification-restriction method including constitutive and polymorphic restriction sites and the use of sequencing in the present study ensures accurate genotyping. This adds to the increasing evidences indicating that several variant *HRH *alleles are actually rarer than initially expected [19,23].

## Conclusion

With regard to PD risk, due the low frequency of the polymorphisms we cannot reach a conclusion of whether HRH polymorphisms can be considered as low-penetrance genes for PD risk with the sample size analyzed. Further studies and/or meta-analyses would elucidate this point. However, besides the low allele frequency observed in patients and control subjects, our findings indicate both *HRH *variant alleles show similar frequencies in both groups, making it unlikely that the studied polymorphisms are major risk factors for the development of PD.

## Competing interests

The author(s) declare that they have no competing interests.

## Authors' contributions

EGM and PA carried out the molecular genetic studies. EGM drafted the manuscript. AL participated in patient's recruitment and clinical evaluation. CM and JAGA participated in the recruitment and clinical evaluation of control subjects. JAGA participated in the design of the study and performed the statistical analysis. EGM and JAGA conceived of the study. All authors participated in the study design and coordination. All authors read and approved the final manuscript.

## Pre-publication history

The pre-publication history for this paper can be accessed here:



## References

[B1] Langlais PJ, Zhang SX, Weilersbacher G, Hough LB, Barke KE (1994). Histamine-mediated neuronal death in a rat model of Wernicke's encephalopathy. J Neurosci Res.

[B2] Thoburn KK, Hough LB, Nalwalk JW, Mischler SA (1994). Histamine-induced modulation of nociceptive responses. Pain.

[B3] Anichtchik OV, Rinne JO, Kalimo H, Panula P (2000). An altered histaminergic innervation of the substantia nigra in Parkinson's disease. Exp Neurol.

[B4] Vizuete ML, Merino M, Venero JL, Santiago M, Cano J, Machado A (2000). Histamine infusion induces a selective dopaminergic neuronal death along with an inflammatory reaction in rat substantia nigra. J Neurochem.

[B5] Coelho MH, Silva IJ, Azevedo MS, Manso CF (1991). Decrease in blood histamine in drug-treated parkinsonian patients. Mol Chem Neuropathol.

[B6] Prell GD, Green JP (1991). Histamine metabolites and pros-methylimidazoleacetic acid in human cerebrospinal fluid. Agents Actions Suppl.

[B7] Agundez JA, Luengo A, Herraez O, Martinez C, Alonso-Navarro H, Jimenez-Jimenez FJ, Garcia-Martin E (2008). Nonsynonymous Polymorphisms of Histamine-Metabolising Enzymes in Patients with Parkinson's Disease. Neuromolecular Med.

[B8] Anichtchik OV, Peitsaro N, Rinne JO, Kalimo H, Panula P (2001). Distribution and modulation of histamine H(3) receptors in basal ganglia and frontal cortex of healthy controls and patients with Parkinson's disease. Neurobiol Dis.

[B9] Molinari SP, Kaminski R, Di Rocco A, Yahr MD (1995). The use of famotidine in the treatment of Parkinson's disease: a pilot study. J Neural Transm Park Dis Dement Sect.

[B10] Gomez-Ramirez J, Johnston TH, Visanji NP, Fox SH, Brotchie JM (2006). Histamine H3 receptor agonists reduce L-dopa-induced chorea, but not dystonia, in the MPTP-lesioned nonhuman primate model of Parkinson's disease. Mov Disord.

[B11] Martinez-Mir MI, Pollard H, Moreau J, Arrang JM, Ruat M, Traiffort E, Schwartz JC, Palacios JM (1990). Three histamine receptors (H1, H2 and H3) visualized in the brain of human and non-human primates. Brain Res.

[B12] Canonaco M, Madeo M, Alo R, Giusi G, Granata T, Carelli A, Canonaco A, Facciolo RM (2005). The histaminergic signaling system exerts a neuroprotective role against neurodegenerative-induced processes in the hamster. J Pharmacol Exp Ther.

[B13] Threlfell S, Cragg SJ, Kallo I, Turi GF, Coen CW, Greenfield SA (2004). Histamine H3 receptors inhibit serotonin release in substantia nigra pars reticulata. J Neurosci.

[B14] Cangioli I, Baldi E, Mannaioni PF, Bucherelli C, Blandina P, Passani MB (2002). Activation of histaminergic H3 receptors in the rat basolateral amygdala improves expression of fear memory and enhances acetylcholine release. Eur J Neurosci.

[B15] Kanamaru M, Iwase M, Homma I (1998). Autoregulation of histamine release in medulla oblongata via H3-receptors in rabbits. Neurosci Res.

[B16] Passani MB, Giannoni P, Bucherelli C, Baldi E, Blandina P (2006). Histamine in the brain: Beyond sleep and memory. Biochem Pharmacol.

[B17] Wiedemann P, Bonisch H, Oerters F, Bruss M (2002). Structure of the human histamine H3 receptor gene (HRH3) and identification of naturally occurring variations. J Neural Transm.

[B18] Mancama D, Arranz MJ, Munro J, Osborne S, Makoff A, Collier D, Kerwin R (2002). Investigation of promoter variants of the histamine 1 and 2 receptors in schizophrenia and clozapine response. Neurosci Lett.

[B19] Sasaki Y, Ihara K, Ahmed S, Yamawaki K, Kusuhara K, Nakayama H, Nishima S, Hara T (2000). Lack of association between atopic asthma and polymorphisms of the histamine H1 receptor, histamine H2 receptor, and histamine N-methyltransferase genes. Immunogenetics.

[B20] SNP linked to Gene HRH1(geneID:3269). http://www.ncbi.nlm.nih.gov/SNP/snp_ref.cgi?locusId=3269&chooseRs=all.

[B21] Orange PR, Heath PR, Wright SR, Ramchand CN, Kolkeiwicz L, Pearson RC (1996). Individuals with schizophrenia have an increased incidence of the H2R649G allele for the histamine H2 receptor gene. Mol Psychiatry.

[B22] Orange PR, Heath PR, Wright SR, Pearson RC (1996). Allelic variations of the human histamine H2 receptor gene. Neuroreport.

[B23] Ito C, Morisset S, Krebs MO, Olie JP, Loo H, Poirier MF, Lannfelt L, Schwartz JC, Arrang JM (2000). Histamine H2 receptor gene variants: lack of association with schizophrenia. Mol Psychiatry.

[B24] SNP linked to Gene HRH2(geneID:3274). http://www.ncbi.nlm.nih.gov/SNP/snp_ref.cgi?locusId=3274&chooseRs=all.

[B25] Hughes AJ, Daniel SE, Kilford L, Lees AJ (1992). Accuracy of clinical diagnosis of idiopathic Parkinson's disease: a clinico-pathological study of 100 cases. J Neurol Neurosurg Psychiatry.

[B26] Agundez JA, Rodriguez I, Olivera M, Ladero JM, Garcia MA, Ribera JM, Benitez J (1997). CYP2D6, NAT2 and CYP2E1 genetic polymorphisms in nonagenarians. Age Ageing.

[B27] Garcia-Martin E, Martinez C, Pizarro RM, Garcia-Gamito FJ, Gullsten H, Raunio H, Agundez JA (2002). CYP3A4 variant alleles in white individuals with low CYP3A4 enzyme activity. Clin Pharmacol Ther.

[B28] Chade AR, Kasten M, Tanner CM (2006). Nongenetic causes of Parkinson's disease. J Neural Transm Suppl.

[B29] Hardy J, Cai H, Cookson MR, Gwinn-Hardy K, Singleton A (2006). Genetics of Parkinson's disease and parkinsonism. Ann Neurol.

[B30] Abeliovich A, Flint Beal M (2006). Parkinsonism genes: culprits and clues. J Neurochem.

[B31] Jimenez-Jimenez FJ, Tabernero C, Mena MA, Garcia de Yebenes J, Garcia de Yebenes MJ, Casarejos MJ, Pardo B, Garcia-Agundez JA, Benitez J, Martinez A (1991). Acute effects of 1-methyl-4-phenyl-1,2,3,6-tetrahydropyridine in a model of rat designated a poor metabolizer of debrisoquine. J Neurochem.

[B32] Maraganore DM, de Andrade M, Lesnick TG, Strain KJ, Farrer MJ, Rocca WA, Pant PV, Frazer KA, Cox DR, Ballinger DG (2005). High-resolution whole-genome association study of Parkinson disease. Am J Hum Genet.

[B33] Clarimon J, Scholz S, Fung HC, Hardy J, Eerola J, Hellstrom O, Chen CM, Wu YR, Tienari PJ, Singleton A (2006). Conflicting results regarding the semaphorin gene (SEMA5A) and the risk for Parkinson disease. Am J Hum Genet.

[B34] Farrer MJ, Haugarvoll K, Ross OA, Stone JT, Milkovic NM, Cobb SA, Whittle AJ, Lincoln SJ, Hulihan MM, Heckman MG, White LR, Aasly JO, Gibson JM, Gosal D, Lynch T, Wszolek ZK, Uitti RJ, Toft M (2006). Genomewide association, Parkinson disease, and PARK10. Am J Hum Genet.

[B35] Goris A, Williams-Gray CH, Foltynie T, Compston DA, Barker RA, Sawcer SJ (2006). No evidence for association with Parkinson disease for 13 single-nucleotide polymorphisms identified by whole-genome association screening. Am J Hum Genet.

[B36] Li Y, Rowland C, Schrodi S, Laird W, Tacey K, Ross D, Leong D, Catanese J, Sninsky J, Grupe A (2006). A case-control association study of the 12 single-nucleotide polymorphisms implicated in Parkinson disease by a recent genome scan. Am J Hum Genet.

[B37] Elbaz A, Nelson LM, Payami H, Ioannidis JP, Fiske BK, Annesi G, Carmine Belin A, Factor SA, Ferrarese C, Hadjigeorgiou GM, Higgins DS, Kawakami H, Kruger R, Marder KS, Mayeux RP, Mellick GD, Nutt JG, Ritz B, Samii A, Tanner CM, Van Broeckhoven C, Van Den Eeden SK, Wirdefeldt K, Zabetian CP, Dehem M, Montimurro JS, Southwick A, Myers RM, Trikalinos TA (2006). Lack of replication of thirteen single-nucleotide polymorphisms implicated in Parkinson's disease: a large-scale international study. Lancet Neurol.

[B38] Dekker MC, Bonifati V, van Duijn CM (2003). Parkinson's disease: piecing together a genetic jigsaw. Brain.

[B39] Bonifati V, Fabrizio E, Vanacore N, De Mari M, Meco G (1995). Familial Parkinson's disease: a clinical genetic analysis. Can J Neurol Sci.

[B40] Elbaz A, Grigoletto F, Baldereschi M, Breteler MM, Manubens-Bertran JM, Lopez-Pousa S, Dartigues JF, Alperovitch A, Tzourio C, Rocca WA (1999). Familial aggregation of Parkinson's disease: a population-based case-control study in Europe. EUROPARKINSON Study Group. Neurology.

[B41] Marder K, Tang MX, Mejia H, Alfaro B, Cote L, Louis E, Groves J, Mayeux R (1996). Risk of Parkinson's disease among first-degree relatives: A community-based study. Neurology.

[B42] de Rijk MC, Tzourio C, Breteler MM, Dartigues JF, Amaducci L, Lopez-Pousa S, Manubens-Bertran JM, Alperovitch A, Rocca WA (1997). Prevalence of parkinsonism and Parkinson's disease in Europe: the EUROPARKINSON Collaborative Study. European Community Concerted Action on the Epidemiology of Parkinson's disease. J Neurol Neurosurg Psychiatry.

[B43] Mancama D, Arranz MJ, Munro J, Makoff A, Kerwin R (2000). The histamine 1 and 2 receptor genes - candidates for schizophrenia and clozapine drug response.. GeneScreen.

[B44] Reference SNP(refSNP) Cluster Report: rs2067470. http://www.ncbi.nlm.nih.gov/SNP/snp_ref.cgi?rs=2067470.

[B45] Garcia-Martin E, Martinez C, Ladero JM, Agundez JA (2006). Interethnic and intraethnic variability of CYP2C8 and CYP2C9 polymorphisms in healthy individuals. Mol Diagn Ther.

[B46] Reference SNP(refSNP) Cluster Report: rs2067474. http://www.ncbi.nlm.nih.gov/SNP/snp_ref.cgi?rs=2067474.

